# Relationship of symptom stress, care needs, social support, and meaning in life to quality of life in patients with heart failure from the acute to chronic stages: a longitudinal study

**DOI:** 10.1186/s12955-021-01885-8

**Published:** 2021-11-06

**Authors:** Min-Hui Liu, Ai-Fu Chiou, Chao-Hung Wang, Wen-Pin Yu, Mei-Hui Lin

**Affiliations:** 1 Heart Failure Research Center, Division of Cardiology, Department of Internal Medicine, Chang Gung Memorial Hospital, 222 Mai Chin Road, Keelung, Taiwan, ROC; 2 Department of Nursing, Keelung Chang Gung Memorial Hospital, Keelung, Taiwan, ROC; 3 School of Nursing, National Yang-Ming Chiao Tung University, No. 155, Sec. 2, Li-Nong St., Taipei, Taiwan, ROC; 4 College of Medicine, Chang Gung University, Taoyuan, Taiwan, ROC; 5 Department of Nursing, Chang Gung University of Science and Technology, Keelung, Taiwan, ROC

**Keywords:** Heart failure, Quality of life, Symptom, Healthcare needs, Meaning in life, Social support

## Abstract

**Background:**

Patients with heart failure (HF) experience continuous changes in symptom distress, care needs, social support, and meaning in life from acute decompensation to chronic phases. The longitudinal relationship between these four factors and quality of life (QOL) was not fully explored.

**Aims:**

To simultaneously investigate the relationship between all factors and QOL from hospitalization to 6 months after discharge, and the impact of the changes in these factors on QOL at different time points.

**Methods:**

A longitudinal design with panel research (4 time points) was used. From January 2017 to December 2019, patients hospitalized due to acute decompensated HF were consecutively enrolled and followed up for 6 months. Patients were interviewed with questionnaires assessing symptom distress, care needs, social support, meaning in life and QOL at hospitalization and 1, 3 and 6 months after discharge.

**Results:**

A total of 184 patients completed 6 months of follow-up. From baseline to 6 months, QOL continuously improved along with decreases in symptoms and care needs, but increases in social support and meaning in life. Better QOL was associated with younger age, higher education level, economic independence, less symptom distress and care needs, and stronger meaning in life (*p* < 0.05). Compared with hospitalization, decreases in care needs and increases in meaning in life at 1, 3 and 6 months were associated with an increase in physical QOL (*p* < 0.01). The decrease in care needs and increase in meaning in life at 3 months were associated with an increase in mental QOL (*p* < 0.05). The increase in social support at 6 months was associated with increases in both physical and mental QOL (*p* < 0.01). Changes in symptom distress were not correlated with changes in QOL from baseline to all time points. In the multivariable analysis, these findings were independent of age, educational level and economic status.

**Conclusions:**

Although symptom distress is associated with QOL after acute decompensated HF, QOL cannot be improved only by improvement in symptoms. With differential duration of improvement in each factor, the integration of alleviation in care needs and strengthening in social support and meaning in life might provide additional benefits in QOL.

## Introduction

Heart failure (HF) is a syndrome derived from various cardiovascular diseases and is a debilitating condition with a limited survival time [1]. In recent years, even with new treatments and advanced surgical modalities to improve outcomes, patients with HF still face the threat of sudden deterioration and unexpected death [2]. HF limits patients’ physiological, psychological and social functions and thus seriously affects their quality of life [3]. In addition, Fotos et al. [4] found that from the perspective of mobility, pain/discomfort, and anxiety/depression, patients with HF have worse quality of life than those with diabetes mellitus, cancer or Alzheimer's disease. Therefore, the goal of HF care is not only to improve the survival rate but also to improve quality of life [5].

Quality of life has been described as a feeling of overall satisfaction and represents the views of individuals on their own life in the context of the culture in which they live, their value of life, and their personal goals, expectations, standards and concerns [6]. At different stages of life, based on the gap between expectations and reality, everyone has different levels of satisfaction with life and develops a sense of quality of life accordingly [6, 7]. Quality of life involves the interaction of multiple factors, including HF-related symptoms [4, 8, 9] care needs [10] and social support [11]. These patients have tremendous care needs to learn how to deal with their physiological limitations and how to adjust their lifestyle to cope with stress and social dysfunction [10]. In addition, the satisfaction of these care needs is closely related to the adequacy of social support.

Previously, through qualitative interviews, Pastor and Moore [12] found that, facing the stress of an incurable disease, patients with HF hovered between living and a sense of dying, questioned the significance of their life, and continually struggled with their reason for living. In addition, Ross and Austin [13] reported that bothered by HF symptoms so severely, they wondered about the value of living. Baldacchino [14] found that the existence of meaning in life can help these patients identify their goals for living and motivate them to learn how to modify their lifestyle. It appears that HF care should also consider the state of meaning in life.

Although the relationships between these factors and quality of life have been investigated in previous studies, most reports investigating care needs and meaning in life were cross-sectional and have focused on patients at the end of life or with terminal stage HF [10, 15, 16]. In addition, previous reports did not extensively investigate quality of life and all of these associated factors in the meantime. Different from the populations in these previous studies, most patients with HF improve gradually after acute decompensation. We hypothesized that, along with substantial improvements in symptom distress, there should be significant decreases in care needs, the development of meaning in life, and the amount of social support. The role of care needs, meaning in life, and social support in quality of life was hypothesized to gradually become insignificant after discharge from acute decompensation. Thus, our study longitudinally and simultaneously collected and analysed all these factors from acute hospitalization to 6 months after discharge. The aims of this study were to clarify the following issues: (1) the changes in quality of life and these four factors from acute hospitalization due to decompensated HF to 6 months after discharge; (2) to simultaneously investigate the longitudinal relationship of demographic characteristics and these factors to quality of life during this 6-month period; and (3) whether there was an impact of the change in these factors on the changes in quality of life and when the impact occurred.

## Methods

### Design

This study and our previous report [17] were derived from the same research project and employed a longitudinal study design (from hospitalization to 6 months after discharge). Our previous study was conducted from January 2017 to May 2019 and recruited 108 patients. The current study was conducted from January 2017 to December 2019. Based on a longitudinal design with panel research (data were collected at four time points), additional 92 patients were enrolled. After enrolment, data collection included structured questionnaires at baseline during acute hospitalization (Time-1) and one month, three months, and six months after discharge (Time-2, Time-3 and Time-4, respectively). Ethics approval was obtained from the institutional review board of the study hospital (IRB No. 201601084B0). This study was conducted in accordance with the principles of the Declaration of Helsinki.

### Participants

Patients were enrolled at a local teaching hospital in Northern Taiwan. The inclusion criteria were as follows: patients who (1) were diagnosed with acute decompensated HF by the cardiologist at hospitalization and had dyspnoea, pulmonary oedema or pleural effusion on chest X-rays (ICD 10: I50.9); (2) had clear consciousness and were able to communicate in Chinese; and (3) were over 18 years old. The exclusion criteria were (1) being bedridden, with an inability to follow the investigators’ instructions; (2) having severe vision and hearing impairment; and (3) suffering from dementia, with an inability to complete the survey by filling out the questionnaires.

**Fig. 1:**
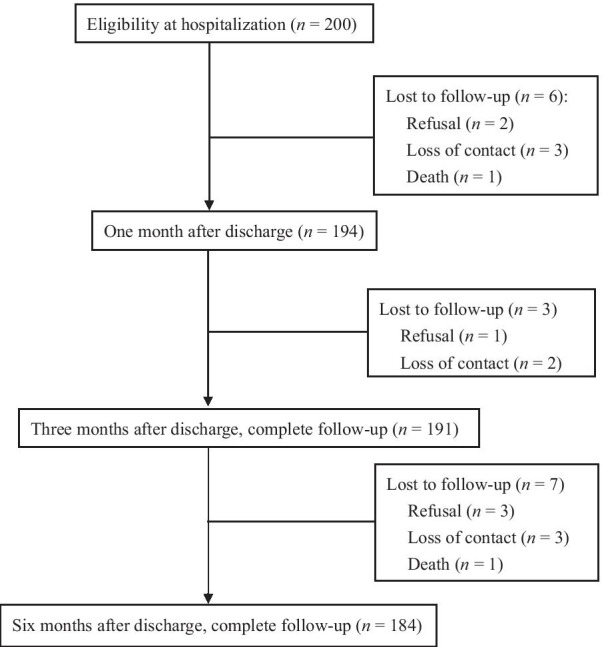
The flow diagram of the study

From January 2017 to December 2019, 200 patients signed informed consent forms, agreed to participate in our study. Of these patients, 16 were lost to follow-up due to death (*n* = 2), refusal (*n* = 6), and loss of contact (*n* = 8). Finally, 184 patients completed the six-month follow-up in our outpatient HF clinic (Fig. 1), for an attrition rate of 8%. The sample size was calculated by G power (University of Kiel, Germany) with *F* test-repeated-measures ANOVA within-factor tests. To achieve 95% power to detect repeated measures of three time points, with an effect size of 0.25 and an alpha of 0.05, a minimum sample size of 132 was required.

### Measures

The questionnaires were administered by a researcher who was an HF nursing specialist and familiar with the instruments to ensure that the same procedure of data collection was followed for all participants. Demographics were collected, including age, gender, education level, marital status, economic status, and living conditions.

Symptom distress was measured using the Chinese version of the symptom distress scale (SDS). The 17-item SDS was used to assess common symptoms in patients with HF. The total score ranges from 17 to 85. A higher score indicates a higher level of symptom distress [18]. In this study, the validity and internal consistency of the SDS were supported by an expert content validity index of 0.8 and a Cronbach’s alpha value of 0.85.

Care needs were measured using the heart failure needs assessment questionnaire (HFNAQ). The 30-item HFNAQ assesses patients’ perceptions of their care needs in the physical, psychological, social, and spiritual domains in the last month. The total possible score ranges from 30 to 150. A higher score indicates greater care needs [19]. We used forward translation and back translation to translate the English version of the HFNAQ into Chinese. In this study, the internal consistency of the Chinese version of the HFNAQ was supported by a Cronbach’s alpha value of 0.85.

Social support was measured using the Chinese version of the Social Support Questionnaire. The questionnaire consists of emotional, appraisal, informational, and tangible support. The total score for each subscale ranges from 0 to 45, with higher scores indicating a higher level of support [20]. The internal consistency of this scale was supported by a Cronbach’s alpha value of 0.95 in this study.

Meaning in life was measured using the Chinese version of the meaning in life questionnaire (MLQ). The MLQ consists of two subscales: the presence of meaning in life (MLQ-P) and the search for meaning in life (MLQ-S). The total score for each subscale ranges from 5 to 35. Higher scores indicate a stronger sense of the presence of meaning in life or search for meaning in life. The Cronbach's alpha values of the original scale ranged from 0.82 to 0.87 for both the MLQ-P and MLQ-S [21]. In this study, the Cronbach’s alpha values were 0.71 and 0.85 for the MLQ-P and MLQ-S, respectively.

Quality of life was measured using the SF-36. This instrument includes two components: the physical component summary (PCS) and mental component summary (MCS). The scores for each subscale range from 0 to 100, with higher scores representing better health outcomes. The internal consistency of this scale was supported by a Cronbach’s alpha value of 0.97 [22]. In this study, the Cronbach’s alpha values were 0.87 and 0.89 for the PCS and MCS, respectively.

### Statistical analyses

The statistical analysis was performed using SPSS 18.0. Independent *t-*tests and chi-square tests were used to examine the homogeneity of participants’ characteristics and outcome variables between the completed and the loss to follow-up groups at baseline/Time-1. Repeated measures ANOVA was used to explore the changes over time in symptom distress, care needs, social support and meaning in life from acute hospitalization to 6 months post discharge. Independent *t*-tests and Pearson’s correlations were used to assess the associations between quality of life and patients’ demographic characteristics, symptom distress, care needs, social support, and meaning in life. Generalized estimating equations (GEEs) were used to identify significant factors associated with changes in quality of life from acute hospitalization to 6 months post discharge. There were no missing data in this study. A two-sided *p* value less than 0.05 indicated statistical significance.

## Results

### Demographic and baseline characteristics

Table 1 presents the characteristics and questionnaire scores at baseline/Time-1 for patients who completed the study (*n* = 184) and those lost to follow-up (*n* = 16). No significant differences in demographic characteristics, symptom distress, social support, care needs, meaning in life or quality of life were noted. The mean age of the 184 patients was 64.4 ± 12.4 years. Most of the patients were male (67.4%), married (66.8%), had an education level lower than high school (72.8%), and lived with family (71.7%). Most of the patients were economically independent (73.4%).

**Table 1:** Homogeneity of participants’ baseline characteristics between the complete follow-up and lost to follow-up groups (*n* = 200)

Team	All (*n* = 200)		Complete (*n* = 184)		Loss (*n* = 16)		*p* value
Variables	*N*	%	*N*	%	*N*	%	
Male	132	66.0	124	67.4	8	50.0	0.159
Education							0.464
Below high school	147	73.5	134	72.8	13	81.3	
Above high school	53	26.5	50	27.2	3	18.8	
Marital status							0.255
Yes	131	65.5	123	66.8	8	50.0	
No	69	34.5	61	33.2	8	50.0	
Economic							0.887
Independent	147	73.5	135	73.4	12	75.0	
Not independent	53	26.5	49	26.6	4	25.0	
Living							0.193
With family	141	70.5	132	71.7	9	56.3	
Alone	59	29.5	52	28.3	7	43.8	

	M	SD	M	SD	M	SD	*p* value
Age	64.9	12.5	64.4	12.4	70.2	12.2	0.075
Symptom distress	41.7	10.5	41.5	10.5	41.2	6.8	0.239
Social support	23.4	11.1	23.3	11.2	24.7	9.9	0.271
Care needs	75.3	14.8	75.2	15.1	73.5	10.2	0.718
Meaning in life							
Presence	24.3	5.0	24.1	5.0	25.5	5.7	0.064
Search	19.8	5.9	19.9	5.9	18.3	5.9	0.311
QOL							
PCS	35.0	10.3	35.2	10.3	32.4	12.0	0.464
MCS	43.5	12.2	43.6	12.2	50.8	11.6	0.900

*QOL* quality of life, *PCS* physical component summary, *MCS* mental component summary

### Factors associated with quality of life at baseline

Factors associated with quality of life were analysed based on PCS and MCS. During hospitalization (baseline/Time-1), factors significantly associated with a better PCS and MCS included age (physical: *r* = − 0.28, *p* < 0.001; mental:* r* = − 0.21, *p* = 0.004), economic independence (physical: *t* = 3.55, *p* < 0.001; mental: *t* = 3.17, *p* = 0.002), less symptom distress (physical: *r* = − 0.46, *p* < 0.001; mental:* r* = − 0.42, *p* < 0.001) and lower care needs (physical: *r* = − 0.47, *p* < 0.001; mental:* r* = − 0.58, *p* < 0.001) (Table 2). Males, compared to females, had a better PCS but a similar MCS. A stronger presence of meaning in life was significantly associated with a better MCS score (*r* = 0.21, *p* = 0.004) but not with PCS. Furthermore, social support and other demographic variables were not correlated with quality of life during hospitalization.

**Table 2:** Relationships of demographic variables and factors to the quality of life in patients with heart failure at baseline (*n* = 184)

Variables	PCS			MCS		
	Mean	SD	*p* value	Mean	SD	*p* value
Gender			0.035			0.110
Male	36.6	10.3		44.6	12.2	
Female	32.9	10.2		41.5	12.1	
Education			0.230			0.230
Below high school	34.6	10.4		42.9	12.5	
Above high school	36.7	10.1		45.4	11.3	
Marital status			0.349			0.658
Yes	36.2	10.4		44.2	12.9	
No	34.7	10.3		43.3	11.8	
Economic			< 0.001			< 0.001
Independence	36.8	10.2		45.2	11.8	
Dependence	30.9	9.4		38.9	12.2	
Living			0.245			0.177
With family	34.6	10.2		42.8	12.0	
Alone	36.7	10.5		45.5	12.6	

	*r*		*p* value	*r*		*p* value
Age	− 0.28		< 0.001	− 0.21		0.004
Symptom distress	− 0.46		< 0.001	− 0.42		< 0.001
Care needs	− 0.47		< 0.001	− 0.58		< 0.001
Social support	− 0.08		0.277	− 0.05		0.480
Meaning in life						
Presence	0.10		0.181	0.21		0.005
Search	− 0.01		0.877	0.11		0.122

PCS: Physical Component Summary of quality of life; MCS: Mental Component Summary of quality of life

### Changes in quality of life and associated factors from hospitalization to 6 months after discharge

**Fig. 2:**
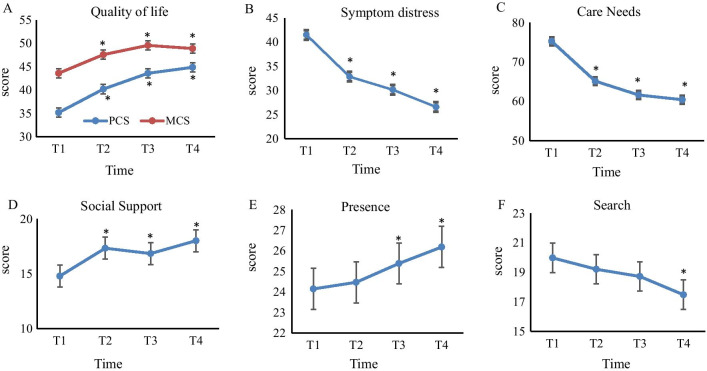
Changes in quality of life (**A**), symptom distress (**B**), care needs (**C**), social support (**D**) and meaning in life (**E** and **F**) from hospitalization to 6 months after discharge; *p < 0.05 compared with the T1

The PCS and MCS scores, compared to the baseline/Time-1, were significantly higher at Time-2, Time-3 and Time-4 (PCS: 36.27 ± 10.35, 40.22 ± 11.11, 43.01 ± 10.62, and 44.88 ± 10.01, respectively, *F* = 63.20, *p* < 0.001; MCS: 43.62 ± 12.39, 47.60 ± 12.06, 49.59 ± 11.37, and 48.91 ± 11.27, respectively, *F* = 17.73,* p* < 0.001) (Fig. 2A). Symptom distress scores were highest at Time-1 and significantly decreased at Time-2, -3 and -4 (41.49 ± 10.55, 32.84 ± 11.02, 30.20 ± 8.27, and 26.72 ± 9.04 at Time-1, -2, -3 and -4, respectively, *F* = 104.35, *p* < 0.001) (Fig. 2B). Care needs scores were also highest at Time-1 (75.28 ± 15.11) and significantly decreased at Time-2, -3 and -4 (65.21 ± 15.43, 61.74 ± 12.37, and 60.55 ± 12.38, respectively, *F* = 78.33, *p* < 0.001) (Fig. 2C). Social support significantly increased from Time-1 to -4 (14.80 ± 8.67, 17.34 ± 7.62, 16.85 ± 7.07, and 18.02 ± 7.85 at Time-1, -2, -3, and -4, respectively, *F* = 7.18, *p* < 0.001) (Fig. 2D). Compared to Time-1, the presence of meaning in life significantly increased at Time-3 and -4 (24.11 ± 5.10, 24.42 ± 5.21, 25.37 ± 5.28, and 26.14 ± 5.34 at Time-1, -2, -3 and -4, respectively, *p* < 0.001) (Fig. 2E); the search for meaning in life significantly decreased at Time-4 (19.96 ± 5.99, 19.20 ± 5.52, 18.71 ± 5.84, and 17.44 ± 6.80 at Time-1, -2, -3 and -4, respectively, *F* = 7.27, *p* < 0.001) (Fig. 2F).

### Factors associated with quality of life among all time points in 6 months

In the univariate analysis based on the GEE model, we investigated the associations of demographic variables, symptom distress, care needs, meaning in life and social support with quality of life among all time points from hospitalization to 6 months after discharge (Table 3). Among the demographic variables, older age (PCS: *B* = − 0.29, *p* < 0.001; MCS: *B* = − 0.21, *p* < 0.001), lower educational level (PCS: *B* = − 3.01, *p* = 0.022; MCS: *B* = − 2.87, *p* = 0.038), and dependent economic status (*B* = − 5.21, *p* < 0.001; MCS: *B* = − 4.14, *p* = 0.011) were associated with poorer physical and mental quality of life, respectively. In addition, we noted that older age was correlated with stronger symptom distress *(B* = 0.13, *p* < 0.001) and more care needs *(B* = 0.30, *p* < 0.001), but less presence of meaning in life (*B* = − 0.09, *p* < 0.001). Lower education level was correlated with more care needs (*B* = − 4.22, *p* = 0.013), but less presence of meaning in life (*B* = − 2.63, *p* < 0.001) and social support (*B* = − 2.23, *p* = 0.027). Economic dependence was associated with increased symptom distress (*B* = 2.89, *p* = 0.009) and care needs (*B* = 4.82, *p* = 0.004), but less meaning in life (*B* = − 1.81, *p* = 0.003).

**Table 3:** Univariate analysis of factors associated with quality of life in patients with heart failure based on the generalized estimating equation analysis model (*n* = 184)

Variables	PCS					MCS				
	B	SE	95%	CI	*p* value	B	SE	95%	CI	*p* value
Age	− 0.29	0.05	− 0.38	− 0.20	< 0.001	− 0.21	0.05	− 0.31	− 0.10	< 0.001
Male	2.08	1.29	− 0.45	4.61	0.107	0.64	1.35	− 2.00	3.29	0.635
Below high school education	− 3.01	1.32	− 5.61	− 0.43	0.022	− 2.87	1.39	− 5.63	− 0.16	0.038
Marriage	− 1.24	1.30	− 3.80	1.32	0.343	− 1.19	1.35	− 3.83	1.45	0.375
Economic dependence	− 5.21	1.40	− 7.96	− 2.46	< 0.001	− 4.14	1.63	− 7.34	− 0.93	0.011
Living with family	− 2.43	1.34	− 5.06	0.19	0.069	− 1.89	1.36	− 4.55	0.76	0.162
Symptoms	− 0.43	0.04	− 0.50	− 0.37	< 0.001	− 0.43	0.05	0.53	− 0.34	< 0.001
T1 * Symptom	Reference									
T2 * symptom	< 0.01	0.08	− 0.16	0.16	0.996	0.04	0.10	− 0.16	0.24	0.720
T3 * symptom	− 0.09	0.08	− 0.25	0.08	0.299	− 0.03	0.12	− 0.26	0.21	0.833
T4 * symptom	− 0.17	0.09	− 0.36	0.01	0.063	0.01	0.12	− 0.23	0.25	0.935
Care needs	− 0.45	0.03	− 0.50	− 0.40	< 0.001	− 0.46	0.07	− 0.59	− 0.33	< 0.001
T1 * care needs	Reference									
T2 * care needs	− 0.15	0.05	− 0.25	− 0.04	0.006	− 0.03	0.07	− 0.17	0.11	0.652
T3 * care needs	− 0.31	0.06	− 0.43	− 0.19	< 0.001	− 0.17	0.08	− 0.32	− 0.01	0.035
T4 * care needs	− 0.27	0.07	− 0.40	− 0.14	< 0.001	0.02	0.09	− 0.15	0.19	0.810
Social support	− 0.02	0.04	− 0.09	0.08	0.966	− 0.04	0.06	− 0.16	0.07	0.441
T1 * Social support	Reference									
T2 * Social support	0.15	0.09	− 0.03	0.32	0.105	0.13	0.09	− 0.04	0.31	0.130
T3 * Social support	0.18	0.10	− 0.02	0.38	0.073	0.17	0.10	− 0.03	0.37	0.087
T4 * Social support	0.31	0.11	0.09	0.52	0.004	0.31	0.11	0.11	0.52	0.003
Meaning in life										
Presence	0.55	0.08	0.39	0.70	< 0.001	0.62	0.11	0.39	0.85	< 0.001
T1 * presence	Reference									
T2 * presence	0.39	0.17	0.06	0.72	0.021	0.18	0.24	− 0.29	0.66	0.454
T3 * presence	0.56	0.15	0.25	0.86	< 0.001	0.49	0.24	0.03	0.95	0.039
T4 * presence	0.71	0.16	0.38	1.03	< 0.001	0.36	0.23	− 0.07	0.81	0.105
Search	− 0.08	0.06	− 0.17	0.07	0.392	− 0.04	0.08	− 0.17	0.13	0.803
T1 * search	Reference									
T2 * search	− 0.09	0.16	− 0.41	0.23	0.584	0.14	0.22	− 0.29	0.56	0.520
T3 * search	− 0.10	0.16	− 0.42	0.22	0.535	0.05	0.21	− 0.36	0.46	0.804
T4 * search	− 0.02	0.15	− 0.32	0.29	0.918	− 0.05	0.19	− 0.44	0.33	0.790

PCS: Physical Component Summary of quality of life; MCS: Mental Component Summary of quality of life

Overall, more symptom distress (PCS: *B* = − 0.43; MCS: *B* = − 0.43, both *p* < 0.001) and care needs (PCS: *B* = − 0.45; MCS: *B* = − 0.46, both *p* < 0.001), and less presence of meaning in life (PCS: *B* = 0.55; MCS: *B* = 0.62, both *p* < 0.001), were significantly correlated with poorer physical and mental dimensions of quality of life, respectively.

### Association between the changes in the factors and the changes in quality of life from baseline to different time points: univariate analysis

In univariate analysis by the GEE model with time interaction, we investigated the impact of the changes in symptom distress, care needs, presence of meaning in life and social support on the physical dimension of quality of life from baseline/Time-1 to 6 months post-discharge (Table 3). Compared with the baseline/Time-1, decreases in care needs (T2: *B* = − 0.15, T3: *B* = − 0.31, T4: *B* = − 0.27, all *p* < 0.01) and increases in the presence of meaning in life (T2: *B* = 0.39, T3: *B* = 0.56, T4: *B* = 0.71, all *p* < 0.05) at Time-2 to Time-4 were significantly associated with increased PCS scores. The increase in social support at Time-4 was significantly associated with an increase in the PCS scores (*B* = 0.31, *p* = 0.004).

For the mental dimension, we also investigated the impact of the changes in symptom distress, care needs, presence meaning in life and social support on quality of life from baseline/Time-1 to 6 months post-discharge (Table 3). Compared with baseline/Time-1, decreases in care needs (T3: *B* = − 0.17, *p* = 0.035) and increases in the presence of meaning in life (T3: *B* = 0.49, *p* = 0.039) at Time-3 were significantly associated with an increase in MCS scores. The increase in social support at Time-4 was significantly associated with an increase in the MCS scores (T4: *B* = 0.31 *p* = 0.003).

Although symptom distress was overall negatively correlated with the PCS and MCS scores, the analysis based on the GEE model with time interaction showed that there were no significant associations between the changes in symptom distress and the changes in PCS and MCS scores from the baseline to any time points after discharge.

### Association between the changes in factors and the changes in quality of life from baseline to different time points: multivariable analysis

In multivariable analysis, these findings remained similar after adjusting for age, educational level and economic status (Table 4). For the physical dimension, compared with the baseline/Time-1, decreases in care needs (T2: *B* = − 0.14, T3: *B* = − 0.28, T4: *B* = − 0.23, all *p* < 0.01) and increases in the presence of meaning in life (T2: *B* = 0.39, T3: *B* = 0.57, T4: *B* = 0.71, all *p* < 0.05) at Time-2 to Time-4 were significantly associated with increased PCS scores. The increase in social support at Time-4 was significantly associated with an increase in the PCS scores (*B* = 0.33, *p* = 0.001). For the mental dimension, compared with baseline/Time-1, decreases in care needs (T3: *B* = − 0.17, *p* = 0.035) and increases in the presence of meaning in life (T3: *B* = 0.49, *p* = 0.036) at Time-3 were significantly associated with an increase in MCS scores. The increase in social support at Time-4 was significantly associated with an increase in the MCS scores (T4: *B* = 0.32, *p* = 0.003).

**Table 4:** Multivariable analysis of factors associated with quality of life in patients with heart failure (over time) based on the generalized estimating equation analysis model (*n* = 184)

Variables	PCS*					MCS*				
Time * Variable	B	SE	95%	CI	*p* value	B	SE	95%	CI	*p* value
			Lower	Upper				Lower	Upper	
Symptoms	− 0.38	0.06	− 0.50	− 0.27	< 0.001	− 0.45	0.08	− 0.62	− 0.29	< 0.001
T1 * symptom	Reference									
T2 * symptom	0.00	0.08	− 0.15	0.15	0.988	0.06	0.10	− 0.13	0.25	0.521
T3 * symptom	− 0.08	0.08	− 0.24	0.08	0.333	0.01	0.11	− 0.21	0.23	0.944
T4 * symptom	− 0.15	0.09	− 0.32	0.03	0.098	0.05	0.12	− 0.18	0.28	0.673
Care needs	− 0.30	0.05	− 0.39	− 0.21	< 0.001	− 0.37	0.05	− 0.46	− 0.29	< 0.001
T1 * care needs	Reference									
T2 * care needs	− 0.14	0.05	− 0.24	− 0.04	0.009	− 0.03	0.07	− 0.17	0.11	0.652
T3 * care needs	− 0.28	0.06	− 0.40	− 0.16	< 0.001	− 0.17	0.08	− 0.31	− 0.01	0.035
T4 * care needs	− 0.23	0.06	− 0.36	− 0.10	< 0.001	0.02	0.09	− 0.15	0.19	0.810
Social support	− 0.15	0.08	− 0.30	− 0.00	0.046	− 0.14	0.10	− 0.34	0.06	0.170
T1 * social support	Reference									
T2 * social support	0.14	0.09	− 0.03	0.32	0.111	0.14	0.09	− 0.04	0.31	0.130
T3 * social support	0.18	0.09	− 0.01	0.37	0.064	0.17	0.10	− 0.03	0.37	0.087
T4 * social support	0.33	0.10	0.13	0.54	0.001	0.32	0.11	0.11	0.52	0.003
Meaning in life	0.51	0.08	0.35	0.66	< 0.001	0.58	0.11	0.30	0.80	< 0.001
T1 * presence	Reference									
T2 * presence	0.39	0.17	0.06	0.72	0.021	0.27	0.24	− 0.20	0.74	0.260
T3 * presence	0.57	0.15	0.28	0.87	< 0.001	0.49	0.23	0.03	0.95	0.036
T4 * presence	0.71	0.16	0.39	1.03	< 0.001	0.41	0.22	− 0.20	0.85	0.062

T1: Hospitalization, T2: 1 month after discharge, T3: 3 months after discharge, T4: 6 months after discharge, PCS: Physical Component Summary, MCS: Mental Component Summary

*Adjusting for age, economic status and education level

In the multivariable analysis, the association between the changes in symptom distress and the changes in PCS and MCS scores remained insignificant from the baseline to any time points after discharge.

## Discussion

After acute decompensation, we noted that the quality of life of patients with HF continuously improved from baseline to 6 months after discharge, along with continuous changes in symptoms, care needs, social support and meaning in life. These factors, age, educational level, and economic dependence were significantly associated with quality of life during the 6-month period. With differential duration of improvement in each factor, the correlation between the changes in care needs, social support and meaning in life and the changes in quality of life from acute decompensation to chronic phase became significantly stronger. However, with time interaction considered, symptom distress became an insignificant factor. These findings were independent of age, educational level and economic status.

Compatible with the previous report by Alaloul et al. [8] based on a cross-sectional design, our cross-sectional analysis revealed that symptom distress was overall associated with quality of life. Interestingly, when time-interaction was considered, the correlation between the changes in symptom distress and the changes in quality of life from acute to chronic phase was not significant. Different from symptom distress, the correlation between the changes in care needs, social support and meaning in life and the change in quality of life was significant and increased from the acute to chronic phase. The increase was noted early in care needs and meaning in life but late in social support. These findings suggest that the quality of life of patients discharged from acute decompensated HF cannot be improved only by improvements in symptoms distress. In addition to symptom distress, efficient interventions with care needs, social support and meaning in life might yield additional benefits in improving quality of life based on different durations of intervention. However, this assumption needs evidence-based support from well-designed interventional studies in the future.

### Meaning in life and quality of life

Previously, Clark et al. [16] demonstrated that although patients with HF continued to live with the stress of death, through spiritual faith, patients could discover their reason for living and the value of life and thereby overcome the threat of unexpected death. Leeming, Murray and Kendall [23] reported that worsening HF caused the loss of independent living ability and patients’ meaning in life followed by loss of happiness and impaired quality of life. Although most studies demonstrated that the stronger meaning in life is, the better patients are able to face the stress of death and the better their quality of life, these studies assessed meaning in life at the end stage of diseases [16, 24, 25]. Our study revealed that the correlation between the presence of meaning in life and quality of life was also present in patients not at the end of life or who did not have advanced HF.

It is interesting to note that meaning in life was already correlated with mental quality of life in facing the acute stress of hospitalization. In addition, similar to our previous report [17], the current study found that meaning in life further increased at 3 months after discharge. Nevertheless, our findings cannot clarify whether the increase in meaning in life at 3 months is due to a transient decrease in response to the influence of worsening HF on pre-existing meaning in life in patients with chronic HF or due to a new development of meaning in life in patients after acute de novo HF. A qualitative interview is warranted to provide further clarification, as it may help clinical professionals to develop efficient interventions. Moreover, the remarkable time-dependent increase in the coefficient of correlation between quality of life and the presence of meaning in life is noteworthy. This notion suggests that appropriate and continuous intervention on meaning in life might lead to a disproportionate increase in quality of life in the chronic phase.

### Social support, care needs and quality of life

A qualitative survey conducted by Wildeboer et al. [26] revealed that stress of sudden death existed in the acute phase and remained in the chronic phases. Comin-Colet et al. [27] reported that patients with HF require continuous and adequate social support for their care and need to be met in a timely manner. In the chronic phase, regardless of whether they return to work or their previous life, patients need to play their role in their social network. Interaction in social networks reduces the fear of loneliness, worry, and stress of living caused by HF [28]. Based on the assessments up to 6 months, our data provided advanced notion that maintenance or even an increase in social support and relief in care needs in the chronic phase were disproportionately associated the improvement in quality of life. Social support and assistance provided by family and medical staff have critical impacts in this regard.

### Demographic characteristics and quality of life

Regarding the demographic variables, our data demonstrated that older age, lower educational level and economic dependence were correlated with poorer quality of life. Erceg et al. [29] noted that elderly individuals are prone to multiple chronic diseases and drug side effects. Their complicated symptoms along with both cardiogenic and noncardiogenic problems are often attributed to notable degeneration and fragility and accordingly lead to poor quality of life. In addition, our data demonstrated that elderly patients and those with low educational level or economic dependence were associated with increased symptom distress and care needs, but less presence of meaning in life and social support. Further investigations in the future are required to determine whether interventions with these factors in specific populations can improve quality of life.

### Limitations

The limitations of our study are as follows. Patients were recruited from one regional teaching hospital using convenience sampling. The data are not representative of the characteristics of the quality of life of patients from different levels of medical institutions. Multicentre studies should be conducted in the future to validate our findings. On the other hand, the participants in this study were those who could communicate and survive for longer than 6 months. Our data could not provide correlations of quality of life with physical, emotional, social and spiritual parameters for patients who were unconscious or exhibited a survival time of less than 6 months. Future studies should adopt a qualitative research design in these specific populations.

## Conclusions

Although symptom distress is associated with QOL after acute decompensated HF, QOL cannot be improved only by improvement in symptoms. With differential duration of improvement in each factor, the integration of alleviation in care needs and strengthening in social support and meaning in life might provide additional benefits in QOL. However, determining whether interventions with respect to these factors in appropriate time windows provide benefits necessitates further evidence-based support from well-designed interventional studies in the future, especially for elderly patients and those with low educational level or economic dependence.

## Data Availability

The datasets used and/or analysed during the current study are available from the corresponding author on reasonable request.

## Abbreviations

HF: Heart failure
QOL: Quality of life
HFNAQ: Heart failure needs assessment questionnaire
SDS: Symptom distress scale
MLQ: Meaning in life questionnaire
LQ-P: Presence of meaning in life
MLQ-S: Search for meaning in life
SF-36: 36-Item Short form survey
